# Retinal Vascular Caliber and Its Ocular and Systemic Correlates in Children Living at High Altitude

**DOI:** 10.3390/children13091263

**Published:** 2026-09-17

**Authors:** Yao Yao, Zhaojun Meng, Lei Li, Weiwei Chen, Jing Fu

**Affiliations:** 1 Beijing Key Laboratory of Ophthalmology & Visual Sciences, Beijing Tongren Eye Center, Beijing Tongren Hospital, Capital Medical University, Beijing 100730, China; stellayao2025@163.com (Y.Y.);; 2 Strabismus and Pediatric Ophthalmology Department, Beijing Tongren Eye Center, Beijing Tongren Hospital, Beijing 100730, China

**Keywords:** retinal vascular diameter, ocular development, children, high altitude

## Abstract

**Highlights:**

**What are the main findings?**

- Longer axial length was associated with smaller CRAE and CRVE, while AVR was positively associated with axial length; these associations were attenuated after axial-length-based ocular magnification correction.
- Lower peripheral oxygen saturation was independently associated with larger CRAE, while myopic children had smaller CRAE and CRVE and higher AVR than non-myopic children.

**What are the implications of the main findings?**

- Retinal vascular caliber is associated with ocular biometric characteristics, refractive status, and peripheral oxygen saturation in children living at high altitude.
- The findings highlight the importance of considering ocular growth, refractive development, and ocular magnification when interpreting retinal vascular measurements in high-altitude children.

**Abstract:**

Background/Objectives: The retina is a highly metabolically active tissue that depends on precise vascular regulation. Childhood represents a critical period of ocular growth, and retinal vascular characteristics may provide insights into the relationship between microvascular development, ocular biometry, and refractive development. However, retinal vascular parameters in children living at high altitude remain poorly characterized. Our goal was to characterize retinal vascular caliber and evaluate its associations with ocular biometric parameters, refractive status, and peripheral oxygen saturation among children living at high altitude. Methods: This cross-sectional baseline analysis included 1410 children from the Lhasa Childhood Eye Study. Retinal vascular parameters, including central retinal arteriolar equivalent (CRAE), central retinal venular equivalent (CRVE), and arteriolar-to-venular ratio (AVR), were measured from fundus photographs using computer-assisted IVAN software. Ocular biometric parameters, cycloplegic refraction, and SpO_2_ were assessed. Multivariable linear regression and sensitivity analyses accounting for school clustering and ocular magnification were performed. Results: The mean age was 7.90 ± 0.49 years, and 47.6% were female. Girls had shorter axial length (AL) and larger CRAE and CRVE than boys. Longer AL was associated with smaller CRAE (B = −5.64, *p* < 0.001) and CRVE (B = −11.24, *p* < 0.001), while AVR was positively associated with AL (*p* = 0.03). Lower SpO_2_ was independently associated with larger CRAE (B = −0.35, *p* = 0.042), but not CRVE. After AL-based relative ocular magnification correction, the associations between AL and CRAE or CRVE were no longer significant. Myopic children had smaller CRAE and CRVE and higher AVR than non-myopic children. Conclusions: Retinal vascular caliber was associated with ocular biometric characteristics, SpO_2_, and refractive status in children living at high altitude. The attenuation of AL-related associations after magnification correction highlights the importance of accounting for ocular magnification in retinal vascular measurements.

## 1. Introduction

The retina is a highly specialized neurosensory tissue essential for visual perception and represents the only human tissue where the microvasculature can be directly and non-invasively visualized. Beyond its fundamental roles in photoreception, signal transduction, and visual information processing, the retina exhibits intense metabolic activity, and its proper function relies on sophisticated blood perfusion and a stable oxygen homeostasis [1,2]. Accordingly, the retina and its vasculature serve not only as the foundation of visual health but also as a vital window reflecting regional tissue metabolism and systemic vascular function [3]. Recent advances in ocular imaging technologies, including fundus photography, optical coherence tomography (OCT), and optical coherence tomography angiography (OCTA), have enabled comprehensive and quantitative characterization of retinal structure and microvascular architecture, establishing retinal imaging as a rapidly expanding field in ophthalmic research. Increasing evidence indicates that retinal vascular alterations are closely linked to the development and progression of ocular diseases and may additionally reflect systemic vascular and metabolic abnormalities.

Childhood represents a critical developmental window during which the visual system undergoes maturation and ocular structures experience substantial remodeling. Unlike mature eyes, pediatric eyes undergo continuous and dynamic growth, accompanied by progressive changes in retinal and choroidal architecture as well as ocular biometric parameters [4]. Establishing normative patterns of retinal structure and vascular development during childhood is therefore fundamental for understanding physiological visual maturation, identifying deviations in ocular growth trajectories, and facilitating early detection and prevention of pediatric eye disorders. The escalating global burden of childhood myopia has further highlighted the importance of pediatric ocular health. Myopia is no longer considered merely a refractive abnormality; rather, it represents a complex developmental disorder characterized by progressive axial elongation, structural alterations of the retina and choroid, and alterations in fundus microcirculation [5]. Investigating retinal vascular development during childhood may provide deeper insights into the biological mechanisms underlying visual development and contribute to the identification of early biomarkers for ocular growth abnormalities and myopia risk.

Retinal vascular development and homeostatic regulation are influenced by a complex interplay of intrinsic and extrinsic factors, including age, sex, ocular biometry, metabolic status, and environmental conditions [5,6]. Among these determinants, oxygen availability plays a pivotal role in maintaining retinal integrity and function. As one of the most metabolically active tissues in the human body, the retina is particularly vulnerable to disturbances in oxygen supply, which may influence retinal vascular regulation, perfusion characteristics, and neurovascular coupling [7]. Therefore, investigating retinal vascular characteristics in children exposed to different oxygen environments may provide valuable insights into the adaptive mechanisms governing retinal microcirculation.

High-altitude environments provide a unique natural model for exploring the impact of chronic hypoxia on pediatric visual system development. Lhasa, located at an average altitude of approximately 3650 m, is characterized by persistent reductions in atmospheric pressure and oxygen availability. Children growing up in this region are exposed to reduced oxygen availability during important periods of ocular development. Chronic exposure to high-altitude environments has been associated with physiological responses including enhanced oxygen transport capacity, altered vascular responsiveness, and metabolic adjustments [8,9]. Although previous studies have extensively investigated the effects of high-altitude exposure on cardiopulmonary function, hematological adaptation, and systemic vascular physiology, the influence of sustained hypoxia on pediatric ocular tissues—particularly the developmental characteristics of the retina and its vasculature—remains poorly understood. It remains unclear whether children living at high altitude exhibit distinct retinal vascular characteristics and whether these characteristics are associated with ocular growth or refractive development [10].

Furthermore, current evidence regarding pediatric retinal vascular characteristics is predominantly derived from populations living at low altitudes, whereas large-scale investigations among high-altitude children remain scarce. Children residing at high altitude may exhibit distinct retinal vascular characteristics due to differences in oxygen availability, environmental exposures, lifestyle, and genetic background. Establishing normative retinal vascular characteristics in high-altitude children and identifying factors associated with vascular development are therefore essential for improving pediatric ocular health assessment and advancing our understanding of visual system development under high-altitude environmental conditions.

Against this background, the present study was conducted within the framework of the Lhasa Childhood Eye Study (LCES). Using standardized fundus photography and computer-assisted retinal vascular analysis, we aimed to systematically characterize retinal vascular morphology in a large cohort of high-altitude children and investigate its associations with ocular biometric parameters, refractive status, and systemic oxygenation. This study seeks to characterize retinal microvascular patterns among children living at high altitude, provide epidemiological evidence regarding the associations between environmental factors and pediatric visual development, and offer a theoretical foundation for childhood eye health surveillance and myopia prevention strategies.

## 2. Materials and Methods

### 2.1. Study Design and Population

The Lhasa Childhood Eye Study (LCES) [11] is a school-based observational cohort study focusing on pediatric ocular health. The study protocol complied with the tenets of the Declaration of Helsinki and was approved by the Ethics Committee of Beijing Tongren Hospital, Capital Medical University (Approval No. TRECKY2019-146). Written informed consent was obtained from the parents or legal guardians of all participating children.

This study adopted stratified cluster sampling to recruit first-grade students in Lhasa, Tibet, China. According to local educational grading standards, 27 of 28 primary schools in Lhasa that participated in the project were categorized into three hierarchical levels. Ultimately, seven primary schools were randomly selected, enrolling a total of 1942 first-grade children. The cohort will be continuously followed up for five years until the participants enter junior high school.

### 2.2. Study Protocols and Examination Methods

All participants received a comprehensive ophthalmic examination, including uncorrected and best-corrected visual acuity (BCVA), stereoscopic vision (S0001, Stereo Optical Co, Chicago, IL, USA), ocular dominance assessment, slit-lamp biomicroscopy (SL-3G, Topcon, Tokyo, Japan), non-contact tonometry (CT-800, Topcon, Tokyo, Japan), ocular alignment evaluation, axial length (AL) (IOL master 500, ZEISS, Oberkochen, Germany), and objective refraction before and after cycloplegia. Fundus photography was also performed for all eligible children.

Refraction was measured before and after cycloplegia using an autorefractor (KR-800, Topcon, Tokyo, Japan), with three consecutive measurements averaged; measurements were repeated if the spherical or cylindrical components differed by more than 0.50 D. After slit-lamp examination to exclude eyes at risk of angle closure, cycloplegia was induced with two drops of 1% cyclopentolate (Alcon, Alcon Inc., Fort Worth, TX, USA) and one drop of Mydrin P (Santen, Santen Pharmaceutical Co., Ltd., Osaka, Japan), administered at 5 min intervals. Thirty minutes after the last drop, a third drop of 1% cyclopentolate was administered if the pupillary light reflex persisted or pupil diameter was <6.0 mm, followed by repeat refraction 15 min later. Participants who did not achieve complete cycloplegia after the third drop were excluded from cycloplegic refraction [12].

Myopia was defined as a spherical equivalent (SE) ≤ −0.50 diopters (D) in the right eye, and hyperopia was defined as SE ≥ +2.00 D in either eye. Anthropometric and physiological measurements included height, weight, and fingertip blood oxygen saturation (SpO_2_) and heart rate. SpO_2_ and heart rate were measured twice using a Digital Pulse Oximeter (Nonin Medical, Inc., Plymouth, MA, USA), and the average values were used for subsequent statistical analyses.

### 2.3. Retinal Vascular Measurements

Monocular 45° color fundus photographs centered on the optic disc (Diabetic Retinopathy Study field 1) and the macula (Diabetic Retinopathy Study field 2) were obtained using a TRC-NW400 fundus camera (Topcon, Tokyo, Japan).

**Figure 1:**
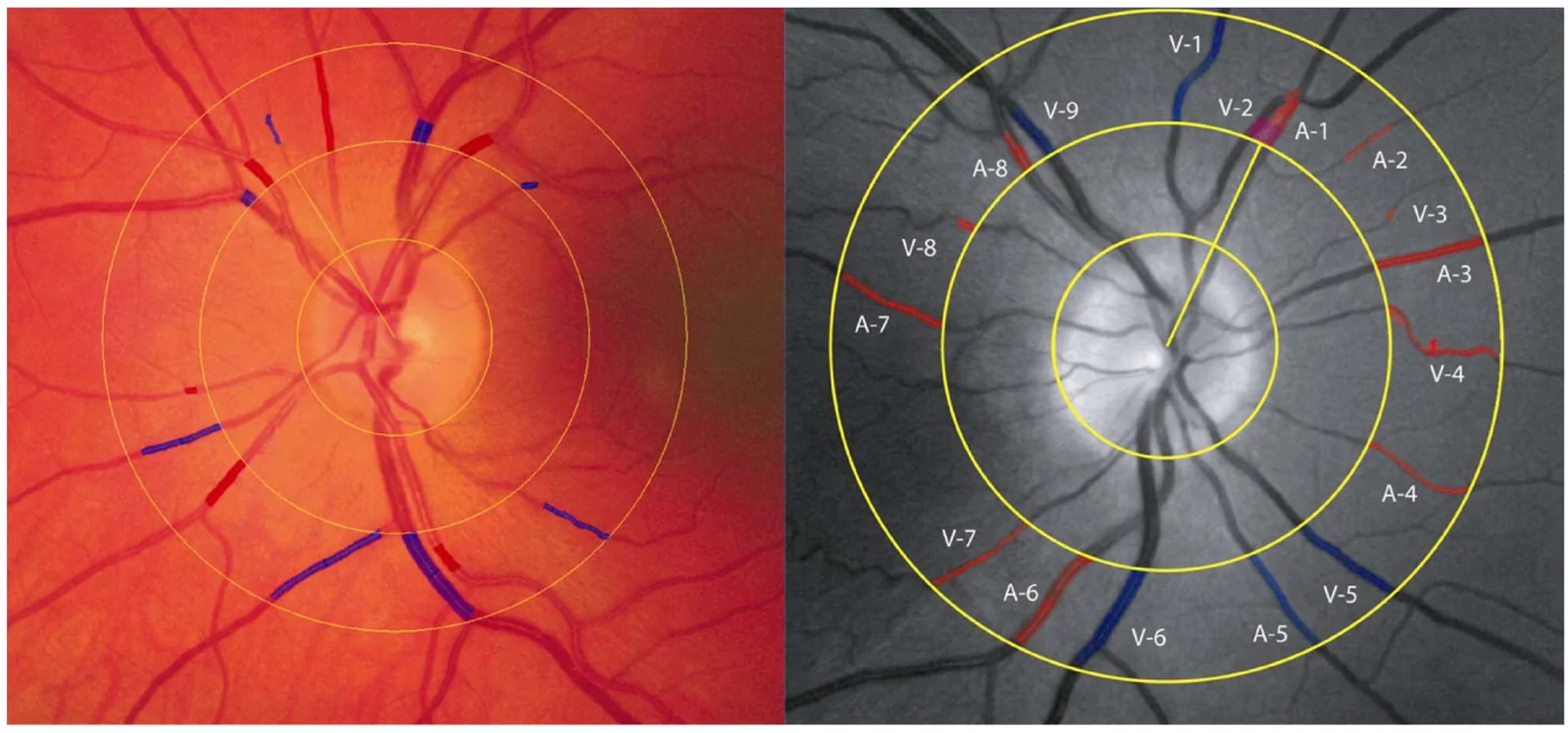
Identification of retinal arterioles and venules in IVAN software.

Retinal vascular calibers were quantitatively assessed using the validated, computer-assisted IVAN software (University of Wisconsin, Madison, WI, USA), according to previously established protocols (Figure 1). The modified Parr–Hubbard formula was used to calculate the central retinal arteriolar equivalent (CRAE) and central retinal venular equivalent (CRVE), and the arteriovenous ratio (AVR) was calculated as CRAE divided by CRVE [13].

For each optic disc-centered fundus photograph, the six largest arterioles and six largest venules within 0.5–1.0 disc diameter from the optic disc margin were identified and measured (Figure 1) [14,15]. Images with poor image quality or unmeasurable major retinal vessels were considered ungradable and excluded from vascular analysis. All vascular measurements were performed by two trained graders who were masked to participants’ demographic and clinical information. Inter-grader reproducibility of retinal vascular measurements was assessed using 400 randomly selected fundus photographs. Two trained ophthalmologists (Yao Y and Meng ZJ), who were masked to participants’ clinical information, independently graded the same images using the IVAN software. The agreement between graders was evaluated using the intraclass correlation coefficient (ICC). Among 400 randomly selected fundus photographs, the ICC for CRAE and CRVE was 0.90 (95%CI: 0.86–0.92) and 0.92 (95%CI: 0.89–0.94). The averaged measurements from the two graders were used for subsequent analyses.

### 2.4. Statistical Analysis

Continuous variables were summarized as mean ± standard deviation (SD), and categorical variables were presented as frequencies and percentages. All statistical analyses were performed using SPSS version 27.0 (IBM Corp., Armonk, NY, USA) and additional statistical procedures for sensitivity analyses. Only right-eye measurements were included in the primary analysis, so that each participant contributed one set of ocular measurements and potential inter-eye correlation was avoided.

Independent-samples *t* tests were used to compare continuous retinal vascular and ocular parameters between boys and girls and between myopic and non-myopic children. Pearson correlation coefficients were calculated to evaluate unadjusted associations between retinal vascular parameters (CRAE, CRVE, and AVR) and ocular and systemic characteristics.

Multivariable linear regression models were subsequently constructed with CRAE, CRVE, or AVR as the dependent variable to evaluate their independent associations with ocular and systemic characteristics. Covariates were selected a priori based on biological plausibility and the objectives of the study rather than solely on statistical significance in univariable analyses. Because axial length (AL), anterior chamber depth (ACD), and spherical equivalent (SE) were strongly correlated, AL was retained as the principal indicator of ocular axial growth to reduce multicollinearity, together with corneal curvature radius. Age, sex, and SpO_2_ were included as prespecified covariates. SpO_2_ was analyzed as a continuous variable in the primary models. Regression coefficients were reported as unstandardized B coefficients, standard errors (SEs), standardized β coefficients, 95% confidence intervals (CIs), and *p* values.

Because participants were recruited through schools, potential within-school correlation was considered in sensitivity analyses. The multivariable associations were re-estimated using linear mixed-effects models with school included as a random intercept. The same prespecified covariates were retained in these models.

Because retinal vascular measurements obtained from fundus photographs may be influenced by ocular magnification associated with axial length, an additional sensitivity analysis was performed using an axial-length-based relative magnification correction. The magnification factor was estimated using the modified Bennett formula [16], q = 0.01306 × (AL − 1.82). A reference axial length of 24.0 mm was used to derive a relative correction factor (q_i_/q_ref_), and corrected CRAE and CRVE were calculated by multiplying the observed measurements by this factor. The relative correction factor was applied multiplicatively because the measured retinal image scale changes in proportion to the ocular magnification factor; using q_i_/q_ref_ therefore rescales each measurement relative to the common reference axial length. Because the camera-specific optical constant required for absolute anatomical correction was unavailable, this procedure was used as a relative sensitivity analysis rather than an absolute anatomical correction [17]. The corrected vascular measurements were subsequently entered into the same school-level mixed-effects models.

All statistical tests were two-sided, and *p* < 0.05 was considered statistically significant.

## 3. Results

### 3.1. Participant Characteristics

A total of 1942 first-grade students from seven primary schools were initially invited. Among them, 1832 participants provided informed consent and underwent examinations. After excluding participants who did not complete the examination procedures (n = 174), those without gradable fundus photographs (n = 16), and those whose images failed automated vascular analysis (n = 232), right-eye fundus images from 1410 participants were included in the final analysis.

The mean age of the 1410 participants was 7.90 ± 0.49 years, 47.6% were female, and 96.3% were Tibetan. The mean cycloplegic spherical equivalent (SE) was 0.51 ± 1.17 D, and the mean axial length (AL) was 22.83 ± 0.81 mm. Mean height, peripheral blood oxygen saturation (SpO_2_), and intraocular pressure were 128.52 ± 5.81 cm, 89.91 ± 3.97%, and 16.36 ± 3.03 mmHg, respectively (Table 1).

### 3.2. Retinal Vascular Parameters and Sex Differences

Sex-stratified analyses showed no significant differences between girls and boys in height, SpO_2_, or cycloplegic SE. Girls had significantly shorter AL and a smaller corneal curvature radius than boys (both *p* < 0.001). In addition, girls had significantly larger CRAE and CRVE than boys (both *p* < 0.01) (Table 1). These findings represent unadjusted between-sex differences, and sex was not independently associated with retinal vascular caliber after multivariable adjustment.

After participants were divided into myopic (164, 11.6%) and non-myopic groups (1246, 88.4%) based on a spherical equivalent cutoff of −0.50 D, compared with non-myopic children, myopic children had significantly smaller CRAE and CRVE (*p* < 0.001 and *p* < 0.01, respectively) and a higher AVR (*p* = 0.04).

### 3.3. Associations Between Retinal Vascular Parameters and Ocular/Systemic Characteristics

Pearson correlation analyses showed that both CRAE and CRVE were negatively correlated with AL (both *p* < 0.001), anterior chamber depth (ACD; *p* = 0.03 and *p* < 0.001, respectively), and corneal curvature radius (*p* = 0.01 and *p* < 0.001, respectively), and positively correlated with cycloplegic SE (both *p* < 0.001). AVR was positively correlated with AL (*p* = 0.03) (Table 2).

Because AL, ACD, and SE were strongly interrelated, AL was retained as the principal ocular growth variable in the multivariable models, together with corneal curvature radius. Age and sex were included as prespecified adjustment variables, and SpO_2_ was entered as a continuous systemic oxygenation measure.

In multivariable linear regression analyses, longer AL was significantly associated with smaller CRAE and CRVE before accounting for potential ocular magnification effects. For CRAE, each 1 mm increase in AL was associated with a 5.64-unit decrease in CRAE (B = −5.64, *p* < 0.001). Similarly, each 1 mm increase in AL was associated with an 11.24-unit decrease in CRVE (B = −11.24, *p* < 0.001). Lower SpO_2_ was also associated with larger CRAE, whereas no significant association between SpO_2_ and CRVE was observed. AVR remained positively associated with AL in the multivariable analysis (Table 3).

### 3.4. Sensitivity Analyses Accounting for School Clustering

Sensitivity analyses using linear mixed-effects models with school as a random intercept yielded similar results. AL remained negatively associated with CRAE (B = −5.63, *p* < 0.001) and CRVE (B = −11.21, *p* < 0.001). SpO_2_ was inversely associated with CRAE (B = −0.353, *p* = 0.042), but not with CRVE (*p* = 0.898). Still, only AL and AVR show a significant correlation (B = 0.02, *p* = 0.02) (Supplementary Table S1).

### 3.5. Sensitivity Analysis for Ocular Magnification

After AL-based relative ocular magnification correction, the association between AL and CRAE was no longer statistically significant (B = 1.80, *p* = 0.100). Similarly, AL was not significantly associated with CRVE after correction (B = −1.19, *p* = 0.321). SpO_2_ remained inversely associated with CRAE (B = −0.331, *p* = 0.043), but not with CRVE (*p* = 0.870). Because CRAE and CRVE were corrected using the same relative magnification factor, AVR was unchanged (Supplementary Table S2).

## 4. Discussion

This study, based on the Lhasa Childhood Eye Study (LCES), systematically assessed the associations of retinal vascular parameters with ocular growth characteristics, refractive status and peripheral oxygen saturation in a large cohort of children residing at high altitude. Our main findings were as follows: (1) girls exhibited shorter AL, smaller corneal curvature radius, and wider retinal arteriolar and venular diameters; (2) both CRAE and CRVE were significantly negatively associated with AL in the primary analyses, whereas AVR was positively associated with AL; (3) SpO_2_ was independently associated with CRAE, while no significant differences in retinal vascular parameters were observed after stratification by oxygen saturation level; and (4) myopic children presented smaller CRAE and CRVE alongside higher AVR. Collectively, these findings indicate that retinal vascular measurements are associated with ocular biometric characteristics and refractive status among children living at high altitude. However, given the absence of a low-altitude comparison group, the cross-sectional design, and the potential influence of ocular magnification on fundus-based measurements, causal interpretations regarding retinal vascular development should be made with caution.

Previous evidence indicates that retinal vessels reflect local microcirculatory status and may be associated with ocular growth processes. Retinal vascular caliber is influenced by multiple factors, including hemodynamic conditions, oxygen metabolic demand, and neuroretinal development. Structural changes accompanying axial elongation may also be reflected in retinal vascular measurements. In the present study, both CRAE and CRVE were significantly negatively associated with AL in the primary analyses, indicating that children with longer AL tended to have smaller measured retinal vascular calibers. This finding is generally consistent with previous pediatric studies. For example, data from the Sydney Childhood Eye Study [18] demonstrated that longer AL was associated with smaller retinal vascular diameters, and similar associations have been reported in studies from Singapore [19].

Several mechanisms may contribute to this association. Axial elongation may alter retinal geometry, tissue stretching, and local vascular distribution, thereby influencing the apparent measurement of retinal vascular caliber. In addition, biological changes associated with ocular growth, including retinal stretching, choroidal alterations, and changes in local hemodynamics, may also contribute. However, because fundus photography-based vascular measurements are affected by ocular magnification, part of the observed association between AL and vascular caliber may reflect measurement scaling effects rather than true anatomical vascular remodeling [20,21]. In our sensitivity analysis incorporating AL-based relative magnification correction, the associations between AL and CRAE/CRVE were attenuated and no longer reached statistical significance, suggesting that ocular magnification should be considered when interpreting relationships between axial length and retinal vascular caliber. Future studies using fully calibrated magnification correction and longitudinal designs are required to clarify whether retinal vascular changes precede or follow axial elongation.

We further identified a positive association between AVR and AL, with children exhibiting longer AL showing higher AVR values. Since CRAE and CRVE are affected by the same relative magnification factor, AVR remains unchanged after this correction and may provide additional information regarding the relative relationship between retinal arteriolar and venular calibers. The observed association may be related to a relatively stronger relationship between venular caliber and AL compared with arteriolar caliber in this cohort. Although the underlying biological mechanisms remain uncertain, differences in vascular regulation between retinal arterioles and venules may contribute. Given the limited evidence regarding AVR and ocular biometric parameters in children, further longitudinal investigations are warranted.

In unadjusted comparisons, girls exhibited larger CRAE and CRVE, accompanied by shorter AL and smaller corneal curvature radius. Previous pediatric studies have reported sex differences in retinal vascular caliber, although the findings have been inconsistent [20,22]. In our cohort, however, sex was not independently associated with retinal vascular parameters after multivariable adjustment. Therefore, the observed differences in retinal vascular caliber between girls and boys may be largely attributable to variations in ocular biometric characteristics rather than a direct effect of sex. Further longitudinal studies are needed to clarify whether sex-related differences influence retinal vascular development during childhood.

Living at high altitude is characterized by reduced atmospheric oxygen availability, and children residing in Lhasa may experience long-term differences in oxygenation environments compared with lowland populations. Experimental studies suggest that oxygen availability can influence retinal vascular regulation through pathways involving hypoxia-inducible factor (HIF) signaling [23]; however, the relevance of these mechanisms in healthy children living at high altitude remains uncertain. Preclinical animal studies have demonstrated that chronic hypoxia triggers retinal vasodilation and upregulates vascular endothelial growth factor (VEGF) expression [24]. Nonetheless, human studies have yielded conflicting results regarding hypoxic effects on retinal vascular caliber. Research in high-altitude adults [9] suggests that acute hypoxia increases retinal blood flow and induces vasodilation, whereas such alterations may gradually attenuate following long-term high-altitude acclimatization [25]. We observed an independent association between lower SpO_2_ and larger CRAE after adjustment for relevant covariates. Although this finding is compatible with the possibility that oxygen availability influences retinal vascular tone, the cross-sectional design does not allow inference of causal mechanisms. This suggests that a single peripheral SpO_2_ measurement may not adequately capture cumulative oxygen exposure or physiological responses related to oxygen availability during childhood. In addition, after adjusting for axial length-related magnification effects and school-level clustering in sensitivity analyses, the major associations between ocular biometric parameters and retinal vascular measures remained consistent, supporting the robustness of the primary findings. Future research incorporating haemoglobin levels, haematocrit and HIF-related biomarkers will enable more comprehensive evaluation of high-altitude hypoxia’s impacts on the retinal microvasculature.

Our study further revealed that myopic children had smaller CRAE and CRVE and higher AVR than non-myopic children. These findings suggest an association between retinal vascular parameters and refractive status, but should be interpreted cautiously given the close relationship between myopia and axial elongation. A growing body of research has explored the roles of retinal and choroidal blood flow changes in myopigenesis [15,18,26]. OCT and OCT angiography studies [27,28] have shown that myopic children frequently demonstrate decreased retinal perfusion density, thinner choroid, and reduced choroidal blood flow. Animal experiments [29,30] further indicate that declines in choroidal blood flow may precede substantial axial elongation in form-deprivation myopia. However, these findings do not establish that reduced retinal vascular caliber is an independent feature or cause of myopia. In our multivariable analyses, axial length was the strongest ocular determinant of retinal vascular caliber, while the associations of axial length with CRAE and CRVE were attenuated after axial-length-based ocular magnification correction. Because the myopia-group comparison was based on uncorrected CRAE and CRVE measurements, the observed differences may therefore partly reflect axial length and ocular magnification rather than an independent effect of refractive status. Thus, these findings should not be interpreted as evidence of a myopia-specific retinal vascular phenotype. AVR is less affected by ocular magnification because the same correction factor applies to CRAE and CRVE and cancels in the ratio. Future studies with individualized magnification correction and longitudinal follow-up are needed to clarify the relationship between retinal vascular characteristics and myopia development.

The present study has several strengths. First, we investigated retinal vascular characteristics in a large cohort of children living at high altitude, providing valuable epidemiological data on pediatric retinal microvasculature under this unique environmental condition. Second, standardized fundus photography and computer-assisted retinal vascular measurements using the IVAN software were applied, and ocular biometric, refractive, and systemic oxygenation parameters were simultaneously considered.

Several limitations should also be acknowledged. First, the cross-sectional design limits causal inference regarding the temporal relationship between retinal vascular measurements, axial growth, and refractive development. Longitudinal follow-up of the LCES cohort will be required to determine whether retinal vascular parameters predict future axial elongation or myopia progression. Second, peripheral oxygen saturation was used as the primary indicator of oxygen status; however, a single SpO_2_ measurement may not fully reflect cumulative hypoxic exposure or physiological adaptation. Future studies incorporating additional markers, such as hemoglobin concentration and hypoxia-related biomarkers, are warranted. Third, although AL-based sensitivity analyses were performed to evaluate ocular magnification effects, residual measurement bias cannot be completely excluded because individualized absolute magnification correction was unavailable. Fourth, we did not have detailed information on behavioral and environmental factors relevant to childhood myopia, including outdoor activity, near-work behavior, and digital-device exposure, which may represent potential residual confounders. Importantly, these behavioral exposures should not be reduced to crude measures such as total screen time alone; their characterization may require information on exposure duration, viewing context, near-work characteristics, outdoor behavior, and other related behavioral domains. More comprehensive assessment of these factors may therefore help to better distinguish the contributions of ocular growth, refractive development, and behavioral environment to retinal vascular characteristics in future studies [31]. Finally, because the present study did not include a low-altitude comparison group, we cannot determine whether the observed vascular characteristics are specific to high-altitude living or reflect general patterns of childhood retinal vascular development.

In conclusion, this study identified significant associations between retinal vascular architecture and ocular development in high-altitude children. Longer axial length and myopia were associated with narrower retinal arterioles and venules, while peripheral oxygen saturation was associated with retinal arteriolar caliber. These findings suggest that retinal vascular parameters may serve as promising biomarkers for evaluating childhood ocular development and myopia risk, and provide epidemiological evidence regarding retinal vascular characteristics and ocular development among children living at high altitude.

## Figures and Tables

**Table 1:** Systemic and ocular parameters of the population studied.

Parameter	Mean	SD	Boys		Girls		*p*-Value
			Mean	SD	Mean	SD	
Age, years	7.90	0.49	7.89	0.44	7.92	0.53	0.45
Height, cm	128.52	5.81	128.50	5.51	128.51	6.00	0.99
Weight, kg	27.28	5.23	27.61	5.39	26.80	4.52	0.045 *
Blood oxygen saturation, %	89.91	3.97	89.76	3.82	90.06	4.11	0.35
Anterior chamber depth, mm	3.54	0.20	3.58	0.21	3.50	0.19	<0.001 *
Axial length, mm	22.83	0.81	23.05	0.78	22.59	0.77	<0.001 *
Corneal radius, mm	7.79	0.26	7.85	0.26	7.72	0.25	<0.001 *
Spherical equivalent, D	0.51	1.17	0.51	1.21	0.55	1.18	0.62
Intraocular pressure, mmHg	16.36	3.03	16.38	3.07	16.41	3.05	0.90
Best-corrected visual acuity	0.02	0.08	0.01	0.06	0.02	0.09	0.08
Central retinal arteriolar equivalent, μm	160.56	14.52	158.98	14.02	162.77	14.87	<0.01 *
Central retinal venular equivalent, μm	216.58	17.13	214.71	16.42	218.64	17.81	<0.01 *
Arteriovenous ratio	0.74	0.08	0.74	0.07	0.75	0.09	0.40

* *p* < 0.05.

**Table 2:** Correlation analysis between systemic and retinal vascular parameters.

Variates	CRAE, μm		CRVE, μm		AVR	
	r	*p* Value	r	*p* Value	r	*p* Value
Age	−0.01	0.77	0.02	0.61	−0.02	0.67
Height	0.06	0.13	0.05	0.20	0.02	0.61
Weight	−0.01	0.85	0.02	0.60	−0.02	0.60
Blood oxygen saturation	−0.08	0.06	−0.02	0.59	−0.06	0.16
Anterior chamber depth	−0.09 *	0.03	−0.22 *	<0.001	0.09 *	0.03
Axial length	−0.25 *	<0.001	−0.40 *	<0.001	0.09 *	0.03
Corneal radius	−0.12 *	0.01	−0.18 *	<0.001	0.03	0.57
Spherical equivalent	0.16 *	<0.001	0.32 *	<0.001	−0.12 *	<0.01
Intraocular pressure	−0.11 *	0.01	−0.08	0.06	−0.04	0.33
Best-corrected visual acuity	−0.04	0.39	−0.12 *	<0.01	0.08	0.06

CRAE, Central retinal arteriolar equivalent; CRVE, Central retinal venular equivalent; AVR, Arteriovenous ratio. * *p* < 0.05.

**Table 3:** Multivariable linear analyses of factors affecting retinal vascular parameters.

Parameters	Unstandardized B	Standard Error	Standardized β	95%CI	*p* Value	VIF
**CRAE, μm**						
Axial length	−5.64	1.14	−0.31	−7.88, −3.40	<0.001 *	2.01
Corneal radius	6.53	3.48	0.12	−0.31, 13.38	0.06	1.96
SpO_2_	−0.35	0.17	−0.09	−0.69, −0.02	0.04 *	1.01
Age	−1.93	1.36	−0.06	−4.60, 0.74	0.16	1.02
sex	1.87	1.39	0.06	−0.87, 4.60	0.18	1.12
**CRVE, μm**						
Axial length	−11.24	1.26	−0.53	−13.72, −8.76	<0.001 *	2.01
Corneal radius	12.73	3.86	0.19	5.15, 20.31	0.001 *	1.96
SpO_2_	−0.03	0.19	−0.01	−0.40, 0.34	0.87	1.01
Age	−0.38	1.50	−0.01	−3.34, 2.57	0.80	1.02
sex	−0.26	1.54	−0.01	−3.29, 2.77	0.87	1.12
**AVR**						
Axial length	0.02	0.01	0.15	0.00, 0.03	0.02 *	2.01
Corneal radius	−0.02	0.02	−0.06	−0.06, 0.02	0.35	1.96
SpO_2_	0.00	0.00	−0.08	0.00, 0.00	0.07	1.01
Age	−0.01	0.01	−0.04	−0.02, 0.01	0.41	1.02
sex	0.01	0.01	0.07	0.00, 0.03	0.13	1.12

CRAE, Central retinal arteriolar equivalent; CRVE, Central retinal venular equivalent; AVR, Arteriovenous ratio; SpO_2,_ blood oxygen saturation. * *p* < 0.05.

## Data Availability

The datasets generated and analysed during the current study are not publicly available due to the personal information of the participants but are available from the corresponding author on reasonable request.

## Supplementary Materials

The following supporting information can be downloaded at: https://www.mdpi.com/article/10.3390/children13091263/s1; Table S1: Sensitivity analyses accounting for school clustering using a linear mixed-effects model; Table S2: Sensitivity analyses for ocular magnification using a linear mixed-effects model.

## Abbreviations

The following abbreviations are used in this manuscript:

CRAE	Central retinal arteriolar equivalent
CRVE	Central retinal venular equivalent
AVR	Arteriovenous ratio
OCTA	Optical coherence tomography angiography
BCVA	Best-corrected visual acuity
AL	Axial length
SE	Spherical equivalent
SpO_2_	Blood oxygen saturation
