# Supplementary material for: Retinal Vascular Caliber and Its Ocular and Systemic Correlates in Children Living at High Altitude
Source: Children (Basel). 2026 Sep 17;13(9):1263. doi: 10.3390/children13091263 (PMC13605173; doi:10.3390/children13091263)
Supplement: Supplementary file 1 [file children-13-01263-s001.zip › children-4524229-supplementary.pdf]

Supplementary Table S1. Sensitivity analyses accounting for school clustering using linear mixed-effects model

Parameters	Unstandardized B	Standard error	Standardized β	95%CI			P value
**CRAE, μm**							
Axial length	-5.63	1.16	-0.31	-7.91	,	-3.35	<0.001
Corneal radius	6.52	3.49	0.12	-0.33	,	13.37	0.06
SpO_2	-0.35	0.17	-0.09	-0.69	,	-0.01	0.04
Age	-1.93	1.37	-0.06	-4.61	,	0.76	0.16
Gender	1.87	1.40	0.06	-0.87	,	4.62	0.18
**CRVE, μm**							
Axial length	-11.21	1.27	-0.53	-13.70	,	-8.71	<0.001
Corneal radius	12.68	3.86	0.19	5.13	,	20.24	0.001
SpO_2	-0.02	0.19	-0.01	-0.40	,	0.35	0.90
Age	-0.35	1.70	-0.01	-3.68	,	2.98	0.84
Gender	-0.27	1.55	-0.01	-3.30	,	2.75	0.86
AVR							
Axial length	0.02	0.01	0.16	0.00		0.03	0.02
Corneal radius	-0.02	0.02	-0.06	-0.06		0.02	0.33
SpO_2	0.00	0.00	-0.08	0.00		0.00	0.08
Age	0.00	0.01	-0.02	-0.02		0.02	0.74
Gender	0.01	0.01	0.08	0.00		0.03	0.09

CRAE, Central retinal arteriolar equivalent; CRVE, Central retinal venular equivalent; AVR, Arteriovenous ratio; SpO_2, blood oxygen saturation.

*P<0.05

Supplementary Table S2. Sensitivity analyses for ocular magnification using linear mixed-effects model

Parameters	Unstandardized B	Standard error	Standardized β	95%CI		P value
**CRAE, μm**						
Axial length	1.80	1.10	0.11	-0.34 ,	3.95	0.10
Corneal radius	6.44	3.29	0.12	-0.01 ,	12.90	0.05
SpO_2	-0.33	0.16	-0.09	-0.65 ,	-0.01	0.04
Age	-1.72	1.30	-0.06	-4.26 ,	0.83	0.19
Gender	1.77	1.32	0.06	-0.81 ,	4.36	0.18
**CRVE, μm**						
Axial length	-1.19	1.20	-0.06	-3.55 ,	1.17	0.32
Corneal radius	12.63	3.65	0.22	5.48 ,	19.78	0.001
SpO_2	-0.03	0.18	-0.01	-0.38 ,	0.32	0.87
Age	-0.28	1.60	-0.01	-3.42 ,	2.87	0.86
Gender	-0.38	1.46	-0.01	-3.24 ,	2.49	0.80

CRAE, Central retinal arteriolar equivalent; CRVE, Central retinal venular equivalent; SpO_2, blood oxygen saturation.

*P<0.05
